# The Immune Protein Calprotectin Impacts Clostridioides difficile Metabolism through Zinc Limitation

**DOI:** 10.1128/mBio.02289-19

**Published:** 2019-11-19

**Authors:** Christopher A. Lopez, William N. Beavers, Andy Weiss, Reece J. Knippel, Joseph P. Zackular, Walter Chazin, Eric P. Skaar

**Affiliations:** a Department of Pathology, Microbiology, and Immunology, Vanderbilt University Medical Center, Nashville, Tennessee, USA; b Vanderbilt Institute for Infection, Immunology, and Inflammation, Vanderbilt University Medical Center, Nashville, Tennessee, USA; c Department of Pathology and Laboratory Medicine, Institute for Immunology, Perelman School of Medicine, University of Pennsylvania, Philadelphia, Pennsylvania, USA; d Department of Chemistry, Vanderbilt University, Nashville, Tennessee, USA; e Department of Biochemistry, Vanderbilt University, Nashville, Tennessee, USA; f Center for Structural Biology, Vanderbilt University, Nashville, Tennessee, USA; g Division of Protective Immunity, Department of Pathology and Laboratory Medicine, Children’s Hospital of Philadelphia, University of Pennsylvania, Philadelphia, Pennsylvania, USA; Baylor College of Medicine; Nanyang Technological University

**Keywords:** *Clostridioides difficile*, Stickland fermentation, calprotectin, proline, zinc

## Abstract

The intestines house a diverse microbiota that must compete for nutrients to survive, but the specific limiting nutrients that control pathogen colonization are not clearly defined. Clostridioides difficile colonization typically requires prior disruption of the microbiota, suggesting that outcompeting commensals for resources is critical to establishing C. difficile infection (CDI). The immune protein calprotectin (CP) is released into the gut lumen during CDI to chelate zinc (Zn) and other essential nutrient metals. Yet, the impact of Zn limitation on C. difficile colonization is unknown. To define C. difficile responses to Zn limitation, we performed RNA sequencing on C. difficile exposed to CP. In medium containing CP, C. difficile upregulated genes involved in metal homeostasis and amino acid metabolism. To identify CP-responsive genes important during infection, we measured the abundance of select C. difficile transcripts in a mouse CDI model relative to expression *in vitro*. Gene transcripts involved in selenium (Se)-dependent proline fermentation increased during infection and in response to CP. Increased proline fermentation gene transcription was dependent on CP Zn binding and proline availability, yet proline fermentation was only enhanced when Se was supplemented. CP-deficient mice could not restrain C. difficile proline fermentation-dependent growth, suggesting that CP-mediated Zn sequestration along with limited Se restricts C. difficile proline fermentation. Overall, these results highlight how C. difficile colonization depends on the availability of multiple nutrients whose abundances are dynamically influenced by the host response.

## Abstract (precis)

Clostridioides difficile infection (CDI) is the leading cause of postantibiotic nosocomial infection. Antibiotic therapy can be successful, yet up to one-third of individuals suffer from recurrent infections. Understanding the mechanisms controlling C. difficile colonization is paramount in designing novel treatments for primary and recurrent CDI. Here, we found that limiting nutrients control C. difficile metabolism during CDI and influence overall pathogen fitness. Specifically, the immune protein CP limits Zn availability and increases transcription of C. difficile genes necessary for proline fermentation. Paradoxically, this leads to reduced C. difficile proline fermentation. This reduced fermentation is due to limited availability of another nutrient required for proline fermentation, Se. Therefore, CP-mediated Zn limitation combined with low Se levels overall reduce C. difficile fitness in the intestines. These results emphasize the complexities of how nutrient availability influences C. difficile colonization and provide insight into critical metabolic processes that drive the pathogen’s growth.

## INTRODUCTION

The lower gastrointestinal tract is colonized by a diverse community of microbes, termed the microbiota, that play critical roles in the normal development and function of the host organism. In turn, the host actively shapes the microbiota structure and function by responding to microbiota-derived signals. Under homeostatic conditions, the host-microbiota relationship facilitates host immune maturation, nutrient acquisition, and resistance to pathogen colonization (1–3). Disruption of the microbiota through antimicrobial compounds, drastic changes in diet (4), or alterations in the host immune status (5) increases susceptibility to colonization by opportunistic pathogens such as Clostridioides difficile. C. difficile infection (CDI) is the leading cause of postantibiotic nosocomial infection and was responsible for approximately 29,000 deaths in the United States in 2011 (6). CDI typically follows antibiotic administration and exposure to C. difficile spores in a hospital setting, although there are increasing incidences of community exposure (6). Subsequent to spore germination into vegetative cells, the C. difficile population expands in the gut and produces potent toxins that are largely responsible for disease and further promote microbial dysbiosis.

During CDI, the gut’s metabolic landscape is dramatically reshaped as a result of the altered microbiota. In human patients, members of the butyrate-producing families *Lachnospiraceae* and *Ruminococcaceae* negatively associate with CDI (7). Correspondingly, butyrate concentrations, along with other short-chain fatty acids (SCFAs), are decreased in CDI patients and are promptly restored following fecal microbiota transfer containing bacteria from the *Lachnospiraceae*, *Clostridiales*, and *Bacteroidetes* (8). SCFAs are by-products of bacterial metabolism that do not inhibit C. difficile directly (9, 10) but reflect the metabolic activities of particular members of the microbiota (11). For instance, mice provided dietary microbiota-accessible carbohydrates experienced shifts in their intestinal microbiota that increased SCFA production and suppressed C. difficile (12).

The microbiota also influences C. difficile adaptation to changes in nutrient abundance. In mouse models of CDI, different antibiotic treatments drive variable changes in the nutrient environment of the gut and concomitant changes in C. difficile gene expression. For instance, treatment with streptomycin increases the relative abundance of the *Firmicutes* during CDI (13) and shifts the C. difficile transcriptome to favor expression of phosphotransferase system (PTS) transporters (14). Conversely, cefoperazone treatment leads to higher abundances of the *Bacteroidetes* (13) and C. difficile expression of ABC sugar transporters and sugar alcohol catabolism genes (14). In conjunction with transcript abundance, over the course of CDI, concentrations of sugar alcohols and other carbohydrates decrease following C. difficile colonization (15). These shifts in C. difficile metabolism are not limited to carbohydrate catabolism, as increases in amino acid fermentation, particularly proline and leucine fermentation, correlate with CDI in mice (14, 15) and in humans (16, 17).

The initial expansion of C. difficile is highly reliant on the nutritional niches opened through disruption of the microbiota; however, soon after colonization, C. difficile toxins trigger a robust inflammatory response that dynamically alters the gut environment. Toxin-induced damage to the gut epithelium allows for translocation of microbe-associated molecular patterns that in turn activate proinflammatory cytokine and chemokine production (18). Neutrophils comprise a large percentage of recruited cells to the infected intestine and aid in controlling C. difficile disease (19). Neutrophils exert their antimicrobial function partly through the production and untargeted release of reactive oxygen species (ROS) (19). However, ROS also react with luminal matter to generate new nutrients, for example, sugar alcohols, that may be used to fuel pathogen growth (14, 20). The impact of the immune response on the availability of nutrients critical to C. difficile growth and survival in the gut remains largely unexplored.

Another facet of the immune response to C. difficile is host nutrient metal restriction, referred to as nutritional immunity (21). As part of this arm of innate defense in response to CDI, infiltrating neutrophils release copious amounts of the protein calprotectin (CP) (22). CP is a common clinical inflammatory biomarker (23), and its expression positively correlates with CDI severity (22, 24, 25). Functionally, CP is a heterodimer of S100A8 and S100A9 (26) that binds and sequesters nutrient metals, including zinc (Zn), manganese (Mn), iron (Fe), and copper (Cu), at two binding sites at the dimer interface (27–29). These metals serve as enzymatic and structural cofactors necessary for the survival of all organisms (30). Thus, CP metal binding limits their availability to bacteria and inhibits pathogen expansion (31, 32). Despite the presence of CP in the intestinal lumen during CDI, C. difficile remains able to persist in the gut. How C. difficile adapts to CP-mediated metal limitation and takes advantage of an inflamed gut environment is not known.

We hypothesize that C. difficile metabolically adapts to dynamic changes in the nutrient landscape of the gut stemming from microbiota dysbiosis and the immune response. In this study, we discovered that the presence of CP leads to global changes in the transcription of an epidemic C. difficile strain, R20291 (NAP1/027). Among the changes was increased expression of genes involved in selenium (Se)-dependent proline fermentation. Zn limitation mediated by CP together with the presence of proline resulted in a synergistic increase in proline fermentation gene transcription. Surprisingly, increased transcription of these genes correlated with lowered proline fermentation efficiency as measured through the by-product of proline reduction, 5-aminovalerate. However, proline fermentation could be restored through supplementation with Se. In mice, low Se availability combined with CP expression and Zn limitation decreased C. difficile proline fermentation-dependent expansion. Overall, these results demonstrate how C. difficile metabolism responds to fluctuations in the availability of multiple nutrients and the consequences on pathogen gut colonization and survival.

## RESULTS

### C. difficile transcriptional analysis in the presence of CP and during infection.

**FIG 1:**
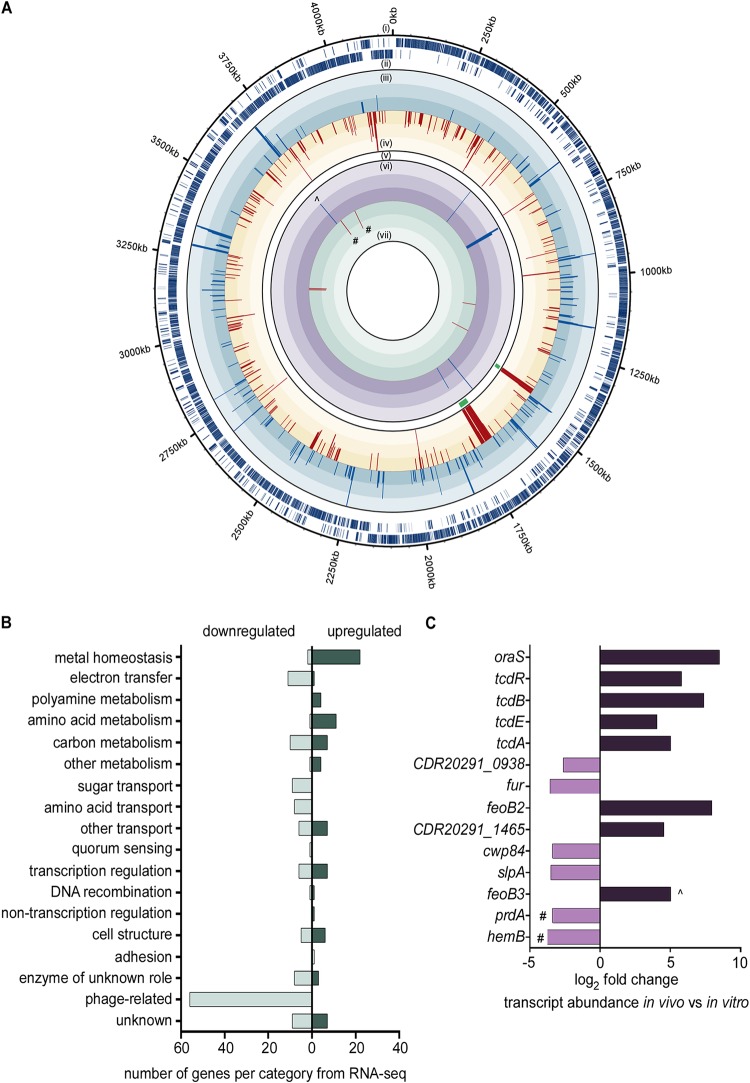
C. difficile alters gene transcription in response to CP treatment and infection. (A) Combined RNA-seq and Nanostring results showing global changes in C. difficile gene expression in response to CP in medium and significantly different changes in gene expression during infection compared to that in medium, respectively. (i and ii) Sense and antisense genes, respectively, in C. difficile strain R20291. (iii and iv) Each bar represents a gene significantly upregulated or downregulated, respectively, in medium containing calprotectin compared to that in medium alone. Each level of color gradation represents a 2.5-fold change. Genes with expression > or <7.5-fold change are represented with bars that hit the maximum limit. (v) Green bars show phage genes present in RNA-seq. (vi and vii) Each bar represents genes from Nanostring panel with significantly upregulated or downregulated expression, respectively, during infection relative to expression in medium. (B) Genes identified in the RNA-seq analysis with significantly different expression grouped into predicted functional categories. (C) Log_2_ fold change of genes from the Nanostring panel significantly upregulated or downregulated during infection compared to expression in medium. #, genes similarly upregulated or downregulated in RNA-seq and Nanostring panel; ^, gene with expression found to be significantly upregulated according to RNA-seq in response to calprotectin but significantly downregulated according to Nanostring during infection.

CP is abundant in the gut lumen during CDI (22), but C. difficile remains able to grow to high numbers. To understand how C. difficile adapts to the presence of CP, C. difficile R20291, an epidemic and hypervirulent strain (33), was grown in medium with or without recombinant human CP. Cells were harvested at early exponential phase (optical density at 600 nm [OD_600_] of 0.3) and RNA was isolated. RNA sequencing of C. difficile transcripts revealed global changes in gene expression when treated with CP compared to transcript abundance in medium alone (Fig. 1A; see also Table S1 in the supplemental material). With CP, 81 transcripts had >3-fold increased expression, while 161 transcripts had 2- to 3-fold increased expression. Expectedly, several genes predicted to be involved in nutrient metal acquisition exhibited some of the highest changes in transcript abundance (Fig. 1B), including a putative Zn transporter (*zupT*), putative ferrous iron transporters (*feo* family), and a gene cluster potentially involved in siderophore uptake (*fhu* operon). Polyamine metabolic genes were also highly upregulated in the presence of CP. These included transporters (*potABCD*) and genes encoding enzymes that convert arginine to agmatine, putrescine, and spermidine (*spe* operon). Also exhibiting modest increases in transcription were genes involved in ethanolamine utilization, necessary for the use of ethanolamine as a carbon and nitrogen source, and allophanate hydrolase, which is involved in urea catabolism. Conversely, 135 transcripts had >3-fold decreased expression, while 230 transcripts had 2- to 3-fold decreased expression. Two phage-related gene clusters exhibited strong downregulation with CP along with the downregulation of genes involved in carbon metabolism, carbohydrate transport, and amino acid transport (Fig. 1B, Table S1). Genes encoding iron-sulfur (Fe-S) cluster-containing proteins were similarly downregulated, likely owing to the redistribution of metals to critical cellular processes.

10.1128/mBio.02289-19.3 TABLE S1 C. difficile genes with significantly different expression when treated with calprotectin. Results from RNA sequencing comparing C. difficile gene expression of bacteria treated with 0.35 mg/ml CP compared to that in medium only. Download Table S1, XLSX file, 0.1 MB. Copyright © 2019 Lopez et al. 2019 Lopez et al. This content is distributed under the terms of the Creative Commons Attribution 4.0 International license.

To narrow the focus on metabolic networks essential to C. difficile colonization, transcript abundances were quantified from C. difficile in a cefoperazone-treated mouse model of infection (34) using Nanostring (Nanostring Technologies, Inc.). Nanostring requires the design of specific capture probes that bind to target gene transcripts followed by binding of a reporter probe containing a barcode sequence of fluorophores unique to the target transcript. The absolute numbers of fluorescent barcodes are then enumerated. The Nanostring gene transcript panel used here consisted of genes upregulated in the CP transcriptome sequencing (RNA-seq) data set along with known virulence genes and global regulators (see Table S2). C. difficile transcript abundances were then compared between bacteria grown in medium and bacteria in cecal content on day 2 postinfection. The primary virulence factors for C. difficile, toxins TcdA and TcdB, were strongly upregulated during infection (Fig. 1C) along with *CDR20291_1465* (putative Mn-containing superoxide dismutase) and *feoB2* (iron transporter). The gene encoding a delta-aminolevulinic acid dehydratase (*hemB*), which is involved in the biosynthesis of siroheme, was expressed at lower levels both during infection and in the presence of CP in medium (Fig. 1C; Tables S1, S2). Interestingly, two genes involved in amino acid metabolism, *oraS* and *prdA*, were strongly induced during infection, with *prdA* also highly upregulated in the presence of CP (Fig. 1C).

10.1128/mBio.02289-19.4 TABLE S2 Target genes and sequences from Nanostring panel. Download Table S2, XLSX file, 0.1 MB. Copyright © 2019 Lopez et al. 2019 Lopez et al. This content is distributed under the terms of the Creative Commons Attribution 4.0 International license.

*prdA* is the first gene in an operon encoding proline reductase, an enzyme that reduces proline to 5-aminovalerate as part of Stickland amino acid fermentation. While *prdA* was the only proline reductase gene included in the Nanostring expression panel, all of the genes encoding proline reductase exhibited increased expression when CP was included in the medium (Table S1). Proline fermentation has been suggested to be a primary metabolic pathway C. difficile uses to balance redox equivalents and produce energy in the form of ATP (35) in both humans (16) and mice (13–15). While expression of the *prd* operon is dependent on the presence of proline (35), other environmental cues that feed into proline fermentation regulation are not known.

### CP-mediated Zn limitation amplifies proline reductase transcription.

**FIG 2:**
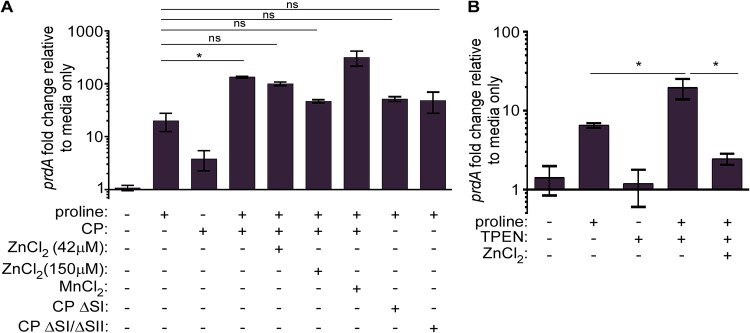
Calprotectin-mediated Zn limitation leads to increased expression of proline fermentation genes. (A) Relative *prdA* expression normalized to *rpoB* via qPCR in TY medium with or without 30 mM l-proline, 42 μM or 150 μM ZnCl_2_, 42 μM MnCl_2_, calprotectin site I mutant (CP ΔSI), or site I and site II (CP ΔSI/SII) mutant. (B) Relative *prdA* expression normalized to *rpoB* via qPCR in TY medium with or without 30 mM l-proline, 75 μM TPEN, or 75 μM ZnCl_2_. *, *P* < 0.05 one-way ANOVA followed by Tukey’s multiple-comparison test; ns, not significant.

Based on the high expression of *prdA* during infection and increased expression of the *prd* operon in the presence of CP, we hypothesized that proline reductase genes are in part regulated by Zn. *In vitro*, *prdA* expression increases in medium supplemented with 30 mM l-proline (Fig. 2A), consistent with literature characterizing the transcriptional regulation of the *prd* operon (36). In response to CP alone, *prdA* transcription also increased, though to a smaller degree than with proline supplementation, suggesting that CP-mediated metal binding alone does not strongly regulate *prd* transcription in the absence of proline. However, when both CP and proline were included in the medium, *prdA* expression was significantly increased compared to that with proline supplementation alone (Fig. 2A).

To determine whether CP Zn sequestration was responsible for the synergistic increase in *prdA* expression, ZnCl_2_ was added at two concentrations to CP-containing medium. Zn supplementation significantly decreased *prdA* expression (Fig. 2A). To test whether this rescue was specific to Zn or a more generalized response to metal limitation, exogenous Mn was added to medium containing CP. Unlike the reduced *prdA* transcription observed when Zn was added, Mn supplementation did not reduce *prdA* expression (Fig. 2A). CP contains two binding sites, site I (SI) and site II (SII), with SI binding multiple metals, including Mn, Zn, Fe, and Cu, and SII binding Zn with high affinity. To ensure CP metal binding, and not the presence of CP, alters *prdA* transcription, CP site mutants (ΔSI and ΔSI/ΔSII) were added to medium together with proline. While *prdA* expression was increased relative to that in medium alone, no synergistic increase in *prdA* expression was observed with either site mutant (Fig. 2A).

As noted above, CP binds multiple nutrient metals. While our data strongly support Zn as the primary metal sequestered by CP that is responsible for changes in *prdA* transcription, we cannot rule out that CP binds other metals that could contribute to a minor degree (Fig. 2A). To enhance specificity toward Zn sequestration, we measured *prdA* transcription in the presence of a chemical Zn chelator, *N*,*N*,*N*′,*N*′-tetrakis(2-pyridinylmethyl)-1,2-ethanediamine (TPEN). Similar to expression with CP, TPEN increased *prdA* expression when proline was added to the medium, and this expression was significantly reduced with Zn supplementation (Fig. 2B). Conversely, the iron chelator dipyridyl did not synergize with proline to enhance *prdA* expression (see Fig. S1). Together, these data support a role for CP-mediated Zn limitation in regulating expression of proline fermentation genes.

10.1128/mBio.02289-19.1 FIG S1 Iron chelation does not increase *prdA* transcription. Relative *prdA* expression normalized to that of *rpoB* via qPCR in TY medium with or without 30 mM l-proline or 50 μM 2,2-dipyridyl. *, *P* < 0.05 by unpaired *t* test. Download FIG S1, TIF file, 0.7 MB. Copyright © 2019 Lopez et al. 2019 Lopez et al. This content is distributed under the terms of the Creative Commons Attribution 4.0 International license.

### Proline fermentation contributes to C. difficile growth during infection.

**FIG 3:**
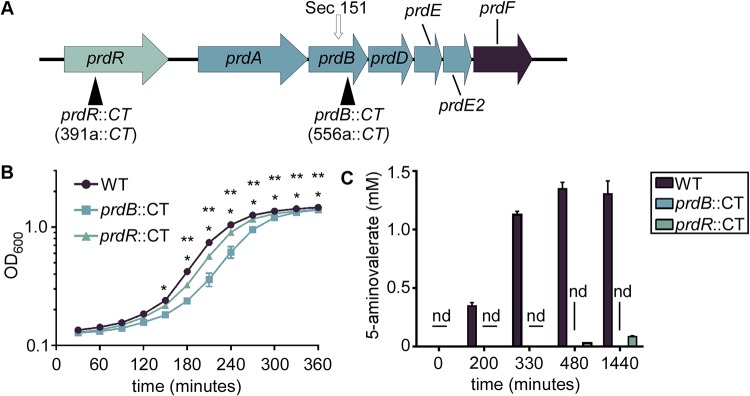
Proline fermentation contributes to C. difficile growth *in vitro*. (A) Genes involved in proline fermentation. Dark triangles indicate location of intron insertion using ClosTron mutagenesis. Arrow indicates the location of the selenocysteine codon in the gene of the main catalytic subunit for the proline reductase, *prdB*. (B) Growth of WT, *prdB*::*CT*, and *prdR*::*CT* strains in TY medium containing 30 mM l-proline as measured by OD_600_. (C) 5-Aminovalerate concentrations in spent media of WT C. difficile, *prdB*::*CT*, and *prdR*::*CT* strains. Error bars indicate standard deviations. nd, not detected; *, *P* < 0.05 between WT and *prdB*::*CT* at indicated time point; **, *P* < 0.05 between WT and *prdR*::*CT* at indicated time point by two-way ANOVA and Dunnett’s multiple comparisons.

C. difficile proline reductase is encoded by 5 genes (*prdABDEE2*) downstream of a transcriptional regulator (*prdR*) that increases expression of the *prd* operon in the presence of proline (Fig. 3A) (36). Also in the operon is *prdF*, encoding a proline racemase that converts l-proline to d-proline for catalysis by the proline reductase (35, 37) (Fig. 3A). To test for the contribution of proline fermentation to C. difficile growth, *prdR* and *prdB*, encoding the catalytic subunit of proline reductase, were disrupted using ClosTron mutagenesis through insertion of a lincomycin resistance intron (*CT*) (38). In tryptone yeast (TY) medium supplemented with 30 mM l-proline, the *prdB*::*CT* mutant strain exhibited significantly lower bacterial density than the isogenic wild-type (WT) strain at equivalent time points. WT C. difficile fermented proline to 5-aminovalerate initiating in early exponential phase, whereas the *prdB*::*CT* strain did not produce 5-aminovalerate (Fig. 3C), confirming that the *prdB*::*CT* strain is deficient in proline fermentation. The *prdR*::*CT* strain remained able to ferment proline, albeit to a lesser degree than the WT strain (Fig. 3C). This resulted in significantly lower bacterial density at equivalent time points *in vitro* (Fig. 3B), though greater than for the *prdB*::*CT* mutant.

**FIG 4:**
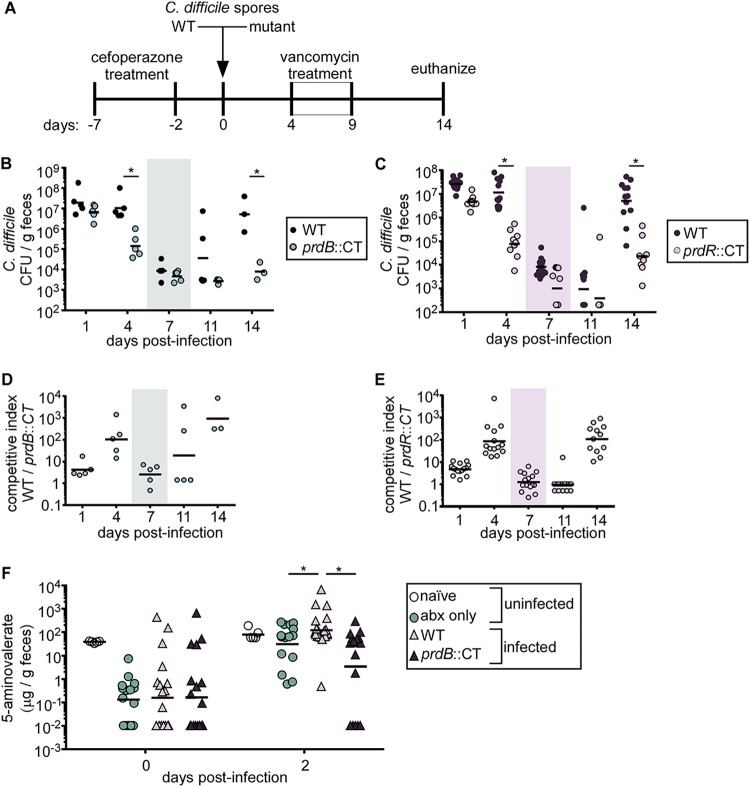
Proline fermentation contributes to C. difficile growth during primary and relapse infection. (A) Model of antibiotic-induced CDI and relapse. (B and C) C. difficile abundances in the feces of mice coinfected with WT C. difficile and the *prdB*::*CT* or *prdR*::*CT* mutant, respectively. Each dot represents CFU from an individual mouse. Shaded boxes represent the times when mice were provided vancomycin to induce relapse. Bars represent geometric means. (D and E) The competitive indices (CIs) (input/output ratios) of WT to mutant C. difficile in mice included in data shown in panels B and C. Each dot represents the CI for an individual mouse. Bars represent the geometric means. (F) 5-Aminovalerate concentrations in the feces of naive mice, mice provided cefoperazone treatment only, or mice provided cefoperazone treatment and subsequently infected with either WT C. difficile or the *prdB*::*CT* mutant. *, *P* < 0.05 by unpaired *t* test.

To ascertain how proline fermentation contributes to C. difficile growth during infection, we used a mouse model of relapsing CDI (39) (Fig. 4A). Mice are treated with cefoperazone in their drinking water prior to inoculation with C. difficile spores. The first 4 days of infection model primary CDI, where C. difficile initially colonizes and expands its population. To model relapse infection, mice are then treated with vancomycin in their drinking water, reducing the pathogen burden to near the limit of detection (approximately 1,000 CFU/g feces). Several days following removal of vancomycin, C. difficile blooms and reinitiates disease. Using this model, mice were inoculated with a 1:1 ratio of WT C. difficile and either the *prdB*::*CT* or *prdR*::*CT* strain. The relative ratio of the two strains was determined by selective plating on taurocholate cycloserine cefoxitin fructose agar (TCCFA) with or without lincomycin. By day 4 postinfection, both mutant strains were recovered in significantly lower numbers than for WT C. difficile (Fig. 4B to E). Following vancomycin treatment, which drastically reduced the overall C. difficile burden, WT C. difficile was recovered at several orders of magnitude higher abundance than for either the *prdB*::*CT* (Fig. 4B and D) or *prdR*::*CT* (Fig. 4C and E) strain.

To further test the hypothesis that C. difficile ferments proline during infection, 5-aminovalerate concentrations were measured from feces of either uninfected mice or mice infected with WT or *prdB*::*CT* C. difficile. Prior to infection but after antibiotic treatment, 5-aminovalerate levels were significantly reduced relative to those in naive mice (Fig. 4F). Two days postinfection, mice infected with WT C. difficile had significantly higher levels of 5-aminovalerate in their feces than either uninfected mice or mice infected with the *prdB*::*CT* strain. These data are consistent with the hypothesis that C. difficile ferments proline *in vivo*. Moreover, 5-aminovalerate levels were similar between uninfected mice and *prdB*::*CT* strain-infected mice (Fig. 4F). Notably, 5-aminovalerate levels from uninfected mice rose from day 0 to day 2 postinfection, indicating that other members of the microbiota ferment proline in the absence of C. difficile. Overall, proline fermentation contributes to C. difficile fitness during both primary and relapsing CDI.

### Se availability influences C. difficile proline fermentation.

**FIG 5:**
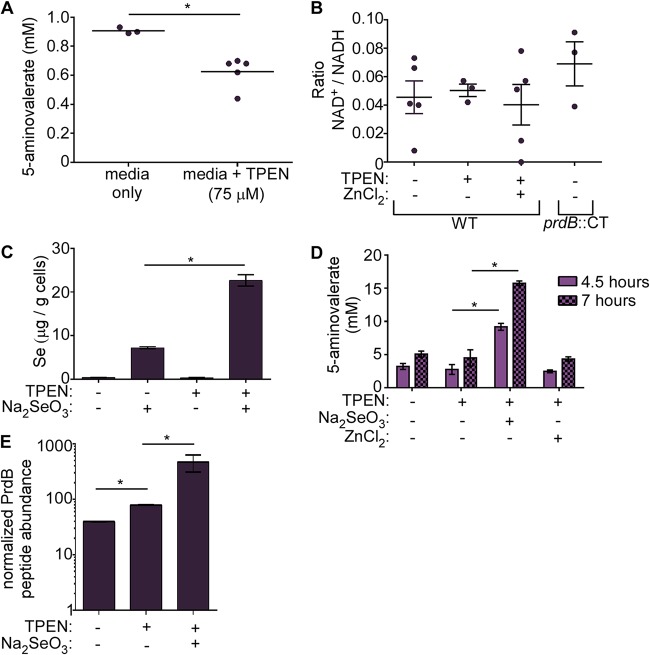
Se and Zn limitation decrease C. difficile proline fermentation. (A) 5-Aminovalerate concentrations in spent medium of C. difficile grown in the presence of 75 μM TPEN. TY was supplemented with l-proline (30 mM final concentration). Each dot represents a single replicate. Bacteria were grown to early exponential phase (OD_600_ of ∼0.2) (B) The intracellular NAD^+^/NADH ratios of WT or *prdB*::*CT* C. difficile grown in TY medium with or without 75 μM TPEN or 75 μM ZnCl_2_. (C) Intracellular selenium abundance in C. difficile grown in TY medium with or without 75 μM TPEN or 10 μM Na_2_SeO_3_ determined through ICP-MS. (D) 5-Aminovalerate concentrations in spent medium from C. difficile grown in TY medium with or without 75 μM TPEN, 10 μM Na_2_SeO_3_, or 75 μM ZnC_l2_ at the indicated time points. Error bars indicate standard deviations. (E) PrdB peptide (TAVIVQR) abundance relative to abundance of an SlpA S-layer peptide (LYNLVNTQLDK). The PrdB peptide is downstream of the selenocysteine residue. Data represent 2 to 3 replicates. Error bars indicate standard deviations. *, *P* < 0.05 by unpaired *t* test.

Our data revealed that genes necessary for proline fermentation were more highly transcribed under Zn-limited conditions and that C. difficile ferments proline to compete in the gut after initial colonization and during relapsing infection. Since C. difficile likely experiences Zn limitation resulting from CP release, we surmised that reduced Zn concentrations provide an environmental signal to shift C. difficile metabolism toward proline fermentation. To test how Zn limitation alters proline fermentation, 5-aminovalerate was measured from C. difficile grown in the presence of TPEN. Surprisingly, TPEN treatment decreased levels of 5-aminovalerate (Fig. 5A) despite increased proline reductase transcription (Fig. 2B). During Stickland fermentation, proline is reduced to replenish NAD^+^, facilitating further oxidation of electron-donating amino acids (35). The lower proline fermentation we observed could be due to an imbalance of the NAD^+^/NADH ratio resulting from disruption of the oxidative branch of Stickland fermentation. However, no significant differences in the NAD^+^/NADH ratios were found between WT C. difficile and TPEN-treated cells or the *prdB*::*CT* mutant (Fig. 5B).

Another factor that could explain decreased proline fermentation despite an increase in gene transcription is Se availability. C. difficile proline reductase is one of only three described C. difficile enzymes that require Se in the form of selenocysteine (Fig. 3A). Therefore, Se deficiency could limit proline fermentation, as selenocysteine is required for the catalytic function of PrdB. To test this possibility, intracellular Se was quantified using inductively coupled plasma mass spectrometry (ICP-MS) from C. difficile grown with or without supplementation with selenite (SeO_3_^2−^), a bioavailable form of Se. We reasoned that if Se was limiting, supplementation would enhance Se uptake by C. difficile. Indeed, selenite supplementation increased intracellular Se that was further increased following treatment with TPEN (Fig. 5C). This suggests that TPEN-mediated Zn limitation drives demand for Se. Selenite supplementation also enhanced proline fermentation under Zn-limited conditions (Fig. 5D), supporting the hypothesis that sufficient Se is required for C. difficile to take advantage of the Zn-dependent transcriptional shift toward proline fermentation.

The discrepancy between transcript levels and proline reduction suggests that there might be a decrease in the proline reductase protein during Se insufficiency. To determine proline reductase protein levels under Zn-limited conditions, PrdB peptide abundance was measured from C. difficile grown in medium with or without TPEN and selenite. While there was a slight, but significant, increase in PrdB with TPEN treatment, selenite supplementation increased PrdB levels nearly 10-fold (Fig. 5E). This difference was not due to differences in abundance of the regulator, PrdR (Fig. S2). Combined, these data reveal that Se abundance is a critical factor for C. difficile proline fermentation under Zn-limited conditions.

10.1128/mBio.02289-19.2 FIG S2 TPEN treatment and Se supplementation do not change PrdR abundance. PrdR peptide (NIGILPVLR) abundance relative to abundance of an SlpA S-layer peptide (LYNLVNTQLDK). Data presented on similar scale as shown in Fig. 5E. Data represent 2 to 3 replicates. Download FIG S2, TIF file, 0.6 MB. Copyright © 2019 Lopez et al. 2019 Lopez et al. This content is distributed under the terms of the Creative Commons Attribution 4.0 International license.

### CP controls C. difficile proline fermentation during infection.

**FIG 6:**
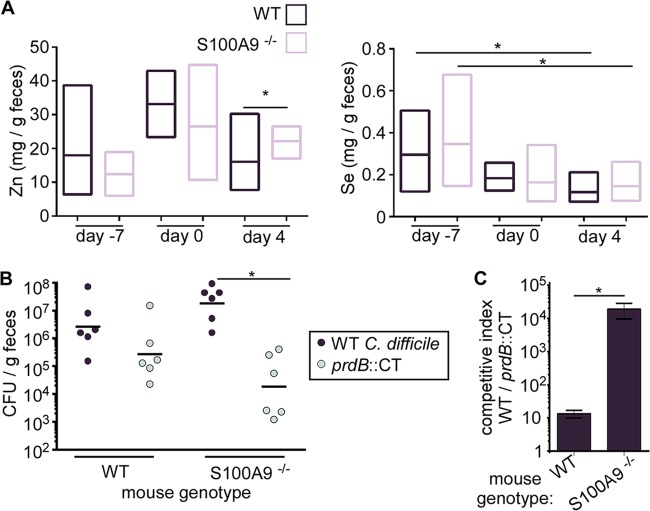
Calprotectin restricts C. difficile proline fermentation during infection. (A) Abundance of Zn and Se in the feces of WT C57BL/6 or S100A9^−/−^ mice infected with WT C. difficile. Box plots represent the median, maximum, and minimum values; *N* = 8 to 10 mice per time point per genotype. (B) C. difficile abundance in coinfected mice at day 2 postinfection. Each dot represents CFU from a single mouse. *, *P* < 0.05, ln-transformed data. (C) The competitive indices of WT C57BL/6 and S100A9^−/−^ mice coinfected with WT and *prdB*::*CT* C. difficile included in the data shown in panel B. *, *P* < 0.05 by unpaired *t* test; error bars represent the standard errors of the means.

C. difficile proline fermentation is controlled by the availability of proline, Zn via CP (Fig. 2 and 5), and Se (Fig. 5). However, it is unclear how these factors contribute to C. difficile fitness during infection. Using the cefoperazone-treated mouse model, concentrations of Zn and Se were measured from the feces of WT C57BL/6 mice and mice lacking CP (S100A9^−/−^) (40) through ICP-MS. Prior to antibiotic treatment and infection (days −7 and 0), levels of both micronutrients were comparable between mouse genotypes (Fig. 6A). After C. difficile colonization and infection, Zn levels were significantly reduced in WT mice compared to that in S100A9^−/−^ mice, confirming a role of CP in limiting Zn availability during CDI. While no genotype-dependent differences were observed in fecal Se, overall Se concentrations were significantly lower during CDI than prior to antibiotic treatment (Fig. 6A), suggesting that Se may be limiting in the gut during CDI.

To discover the role of CP in C. difficile proline fermentation-dependent fitness, WT and S100A9^−/−^ mice were infected with a mixed inoculum of WT C. difficile and the *prdB*::*CT* mutant. At day 2 postinfection, the WT C. difficile strain was recovered at approximately 10-fold higher levels than the *prdB*::*CT* strain (Fig. 6B and C). On the other hand, in S100A9^−/−^ mice, WT C. difficile outcompeted the *prdB*::*CT* strain by roughly 4 orders of magnitude (Fig. 6C). Together with the quantified fecal Zn and Se, these data support a model whereby CP controls the proline fermentation-dependent expansion of C. difficile in the Se-limited gut environment by limiting Zn.

## DISCUSSION

CDI consists of interactions between the microbiota, host response, and pathogen that are influenced by environmental factors such as antibiotics or diet. An important node within these interactions is the metabolism of C. difficile, which is intricately connected to virulence and growth. An examination of the C. difficile genomic repertoire reveals a high capacity for multiple energy- or redox-balancing metabolic pathways, particularly in carbohydrate and amino acid metabolism (41, 42). While there is great heterogeneity among C. difficile isolates, there exists significant overlap in the capacity to metabolize nutrients potentially important during infection (33). Yet, the specific metabolic pathways important to C. difficile during infection, or how flux through those pathways shifts in a dynamic gut environment, are not entirely known.

A dominant driver in reshaping the nutrient profile of the gut is the immune response to infection. In particular, nutritional immunity to control pathogen growth by limiting access to metals impacts pathogen colonization of the gut (43–47) and systemic sites (48–50). Many of the studies that analyze CP in nutritional immunity focus on how Zn or Mn limitation changes bacterial expression of metal transporters or the function of metal-dependent enzymes such as Mn-dependent superoxide dismutase and catalase. Less described is how pathogens metabolically respond to changes in nutrient metal availability and the consequences on colonization or expansion in the gut.

To address these questions, we combined an *in vitro* RNA-seq approach with a targeted analysis of C. difficile transcript abundance *in vivo*. Genes involved in proline fermentation were among the highest upregulated in response to CP *in vitro* and were significantly increased in abundance during infection (Fig. 1). Recent metatranscriptomic and metabolomic studies hypothesized C. difficile proline fermentation is a dominant metabolic pathway critical to gut survival (13–15). Later studies showed that proline fermentation contributed to C. difficile colonization in gnotobiotic mice transplanted with feces from human donors with dysbiotic microbiota (16). While these data provide valuable insight into the role of proline fermentation in C. difficile colonization, associations between C. difficile metabolism and the immune response are lacking. Here, transcriptional changes in the presence of CP suggest that host control of nutrient metals directs this critical metabolic process.

Further analysis revealed that Zn, and not Mn, limitation in the presence of proline synergizes to enhance proline reductase gene transcription (Fig. 2A). Previous studies found that proline is necessary for high *prd* operon transcription and that this is dependent on the regulator PrdR (36). Congruently, PrdR represses other NAD^+^-generating pathways, such as glycine fermentation, indirectly via the redox-responsive regulator Rex (51). Our data indicate that another layer of regulation overlaps with known regulatory pathways that are dependent on Zn availability. A possible candidate regulator found in bacteria is the Zn uptake regulator (Zur); however, no Zur proteins have been identified in C. difficile. A related protein, ferric uptake regulator (Fur), mediates bacterial responses to Fe availability, typically through repression under iron-replete conditions, and requires Zn for protein structure (52). C. difficile encodes Fur and a putative Fur-box is found upstream of *prdA* (data not shown) with sequence similarity to known Fur-regulated genes (53). Additionally, a recent study found increased proline reductase gene transcripts in a *fur* mutant, though no difference at the protein level was described (54). While it is conceivable that Zn availability may regulate Fur activity by preventing Fur dimerization in the absence of structural Zn, several lines of evidence argue against Fur playing a role. Using an Fe-specific chelator, we found no synergism in *prdA* transcription when iron was limiting in the medium (Fig. 2C). Also, the putative Fur box is located approximately 130 bp upstream of the −10 and −35 sigma factor binding sites of *prdA*. As a repressor, Fur-boxes typically overlap these binding sites (53, 55). Finally, microarray analysis of a C. difficile *fur* mutant found no difference in proline reductase gene expression (53). Therefore, the mechanism connecting Zn limitation to enhanced *prd* operon transcription remains elusive.

Proline fermentation contributes to the growth of epidemic and hypervirulent C. difficile R20291 *in vitro* (Fig. 3B) and during infection (Fig. 4), though the exact role of proline reduction to overall C. difficile physiology is unknown. In Clostridium sporogenes, proline reduction is coupled to the generation of a proton motive force for the downstream production of ATP (56), a function not yet demonstrated in C. difficile. More likely, proline reduction primarily regenerates NAD^+^ or other reducing equivalents for further amino acid oxidation in Stickland fermentation that, depending on the amino acid donor, can generate ATP. A similar principle was shown for the enteric pathogen Salmonella enterica, where a redox-balancing reaction was favored over direct ATP production via nitrate reduction (57). Regardless, proline fermentation provides a fitness advantage both during primary infection and during outgrowth in relapse (Fig. 4B to D). Further evidence of this was shown through measurements of 5-aminovalerate from murine feces, where infection with WT C. difficile resulted in higher 5-aminovalerate concentrations than in mice infected with a *prdB*::*CT* mutant (Fig. 4E). Of note, 5-aminovalerate concentrations were increased in both uninfected mice and *prdB*::*CT* strain-infected mice compared to that day 2 postantibiotic treatment (day 0), suggesting the microbiota ferments proline and that competition for proline could dictate C. difficile-microbe interactions. It is tempting to speculate that disruption of the microbiota through antibiotics or other factors temporarily opens a proline-rich niche for C. difficile, allowing the pathogen access to a valuable nutrient early during colonization. Furthermore, the source of proline may change during the course of infection, being initially diet derived (16) early during colonization and possibly host-derived in the form of proline-rich collagen as infection progresses or during relapse. How proline availability changes during infection will be an interesting field of future inquiry.

Proline reductase is a selenoenzyme, harboring selenocysteine in PrdB through a modification of the canonical UGA stop codon (58). While Zn limitation and proline synergize to increase proline reductase transcription (Fig. 2), the abundance of the PrdB peptide is not fully realized in the absence of sufficient Se (Fig. 5E). This then limits proline fermentation efficiency (Fig. 5A and D) through an unknown mechanism. C. difficile can grow in media without Se, but it is unclear why a critical process such as proline fermentation is dependent on this trace element. It is important to note that even small amounts of Se can be incorporated into functional PrdB for fermentation (Fig. 5D). Additionally, the other two Se-containing enzymes, GrdA and GrdB from glycine reductase, are downregulated when proline is fermented (36, 51). This would allow all available Se to be channeled toward incorporation into PrdB. Lastly C. difficile proline fermentation only requires one selenoenzyme. In contrast, Clostridium sticklandii, the model for Stickland amino acid fermentation, contains another selenocysteine containing enzyme, PrdC (59). PrdC reduces PrdB following catalytic reduction of proline, regenerating proline reductase for further activity (59). C. difficile PrdC likely serves the same function as that in *C. sticklandii* but contains a cysteine residue in place of selenocysteine. Again, this may be an adaptation to minimize the demand for Se and incorporate even trace amounts of the element into the optimal metabolic machinery.

During infection, CP limits proline fermentation-dependent C. difficile growth (Fig. 6B and C), and this inhibition is associated with both Zn and Se limitation (Fig. 6A). Importantly, the data presented only provide insight into how CP affects C. difficile proline fermentation and do not address how CP affects other energy-producing metabolic pathways. However, we propose three possible explanations for why C. difficile proline fermentation is linked to CP expression that are not mutually exclusive. First, proline reductase activity is inhibited by high concentrations of Zn (35); thus, proline reductase activity may be optimal when Zn is deplete. In this way, the host defense actually benefits C. difficile outgrowth. A second explanation is that CP-dependent Zn limitation generally signals an immune response. The presence of CP causes global shifts in C. difficile transcription (Fig. 1A) that could indicate adjustment to an inflamed gut environment with altered nutrient availability and microbial competition. For instance, ethanolamine derived from host cell membranes during inflammation is used as nitrogen and carbon sources for the enteric pathogens S. enterica and Escherichia coli (60, 61) to outcompete the microbiota. C. difficile genes for ethanolamine utilization were increased with CP (see Table S1 in the supplemental material), potentially indicative of a situation when inflammation results in increased ethanolamine availability. Proline fermentation then may be only part of a broader adaptation to a pathogenic lifestyle. Third, it is conceivable that CP only lowers C. difficile proline fermentation-dependent growth when Se is limiting. Increased dietary Se intake or decreased host Se absorption could drastically alter CP’s protective function to favor C. difficile proline fermentation. Therefore, the relative benefit of C. difficile proline fermentation is highly contextual and dependent on the dynamic availability of Zn and Se.

C. difficile harbors numerous metabolic pathways that likely contribute to the overall growth and survival of the C. difficile population during infection. Here, we found that proline fermentation contributes to C. difficile outgrowth in the gut and is regulated by the immune response and nutrient availability. Further defining the dominant metabolic networks and determining how those networks are regulated are important steps in ultimately designing strategies that target C. difficile metabolism and combat CDI.

## MATERIALS AND METHODS

### Bacterial strains and culture conditions.

For the bacterial strains used in this study, see Table 1. Escherichia coli strains were routinely cultured aerobically in lysogeny broth (LB) or agar (10 g/liter tryptone, 5 g/liter yeast extract, 10 g/liter NaCl) with the appropriate antibiotic. Bacillus subtilis strains were routinely cultured aerobically in LB or brain heart infusion (BHI) medium (52 g/liter BHI) with the appropriate antibiotic. C. difficile was routinely cultured anaerobically in brain heart infusion medium supplemented with yeast extract (BHIS) (52 g/liter BHI, 5 g/liter yeast extract, 0.03% l-cysteine) (62) in an atmosphere of 5% CO_2_, 5% H_2_, and 90% N_2_. Antibiotic concentrations were used as follows unless otherwise noted: kanamycin (Kan), 50 μg/ml; carbenicillin (Carb), 100 μg/ml; chloramphenicol (Cm), 30 μg/ml or 2.5 μg/ml; tetracycline (Tet), 5 μg/ml; lincomycin (Linc), 20 μg/ml; thiamphenicol (Tm), 20 μg/ml.

**TABLE 1:** Bacterial strains and plasmids used in this study

Strain or plasmid	Relevant information	Reference or source
Strains		
Clostridioides difficile strain R20291	Ribotype 027	33
C. difficile *prdR*::*CT*	ClosTron intron inserted after nucleotide 391 in *prdR* (*prdR* 391a::*CT*), Linc^r^	This study
C. difficile *prdB*::*CT*	ClosTron intron inserted after nucleotide 556 in *prdB* (*prdB* 556a::*CT*), Linc^r^	This study
Bacillus subtilis JH BS2	Tn*196*, Tet^R^	64
Escherichia coli DH5α	F^−^ *endA1 glnV44 thi-1 recA1 relA1 gyrA96 deoR nupG* ϕ80d*lac*ΔM15 Δ(*lacZYA*-*argF*)*U169 hsdR17*(r_K_^−^ m_K_^+^) λ^2212^	64
E. coli MG1655	RecA^+^	63
Plasmids		64
pCR-Blunt	Cloning vector, Kan^r^	Thermo Fisher
pJS107	Tn*916*, *oriT*	64
pCAL127	pJS107_*prdR* intron	This study
pCAL128	pJS107_*prdB* intron	This study
pJS116	pCD6 ColE1 Tn*916 oriT*, Cm^r^	64

### Cloning and mutant generation.

For *prdR*::*CT* and *prdB*::*CT* strain generation, polar mutations were created using the ClosTron system as described previously (38). Here, gBlocks containing sequence modifications to target *prdR* and *prdB* in C. difficile strain R20291 were generated using the TargeTronics algorithm (TargeTronics, LLC, Austin, TX) and synthesized by Integrated DNA Technologies. The gBlocks were cloned into pCR-Blunt using the Zero Blunt pCR cloning kit (Thermo Fisher). The gBlock fragment was excised from pCR-Blunt by digestion with HindIII and BsrGI (NEB) and ligated (NEB T4 ligase) into pJS107 linearized using the same restriction enzymes. DH5α E. coli cells were transformed with the ligated plasmids via heat shock. Following screening to confirm plasmids contained the correct insert, pCAL127 and pCAL128 were then transferred to E. coli MG1655 (63) via heat shock to prepare for B. subtilis transformation. B. subtilis JH BS2 (64) was incubated at 30°C on a plate of LB plus 2.5 μg/ml Cm (Cm_2.5_) overnight. On the day of transformation, the recipient B. subtilis strain from the plate was used to inoculate 10 ml of SpC medium {0.1 ml 50% glucose, 0.1 ml 2% MgSO_4_, 0.25 ml 1% Casamino Acids, 0.2 ml 10% nutrient broth, 0.05 ml 1% tryptophan, 9.3 ml T base [2 g/liter (NH_4_)_2_SO_4_, 14 g/liter K_2_HPO_4_, 6 g/liter KH_2_PO_4_, 1 g/liter Na_3_-citrate·6H_2_O]} and shaken at 280 rpm at 37°C until they reached early stationary phase. The culture was then diluted 1:5 into 10 ml of SpT medium (0.1 ml 50% glucose, 0.41 ml 2% MgSO_4_, 0.1 ml 1% Casamino Acids, 0.1 ml 10% nutrient broth, 0.05 ml 1% tryptophan, 9.24 ml T base) and shaken for 70 min at 250 rpm at 37°C. Following incubation, 1 ml of B. subtilis was transferred to a fresh tube and inoculated with 1 μl pCAL127 or pCAL128 plasmid prep and shaken at 37°C for 30 min. The cells were pelleted by low-speed centrifugation and then resuspended in 1 ml LB. Transformed cells were then incubated shaking for 90 min to recover, followed by plating on LB plus Cm_2.5_ plus Tet.

To transfer pCAL127 and pCAL128 to C. difficile, B. subtilis with the respective plasmids was grown overnight on LB plus Cm_2.5_ plus Tet, and C. difficile was grown overnight in BHIS broth. The C. difficile culture was then diluted 1:20 in fresh BHIS broth and incubated at 37°C for 6 h. Meanwhile, 5 ml of BHI broth plus Cm_2.5_ and Tet was inoculated with B. subtilis taken from the plates using a loop and grown shaking at 37°C for 6 h. After incubation, the B. subtilis cultures were moved into the anaerobe chamber, and 100 μl was spotted onto a BHIS plate. Next, 100 μl of the C. difficile culture was spotted on the other side of the same BHIS plate. Each spot was spread independently for a few seconds before being combined to allow some of the antibiotics to absorb into the agar before contacting C. difficile. The conjugation plate was then incubated for 24 h at 37°C. Following incubation, 3 ml of BHIS broth was added to each plate to resuspend the bacteria, which were then transferred to a clean tube. These bacteria were then plated on BHIS plus Tm plus Kan to select for C. difficile that took up the plasmids. C. difficile was then checked for sensitivity to Tet (to confirm loss of the Tn*916* transposon) and resistance to Linc (to confirm insertion of the intron). Potential mutant strains were confirmed via PCR using primers listed in Table 2.

**TABLE 2:** Oligonucleotide sequences used in this study

Name	Sequence (5′→3′)^*a*^	Reference
Primers		
*prdA*-qPCR-FW	**GGTCAAGTACTAGGAGCTAAGT**	This study
*prdA*-qPCR-RV	**CTACTTCTTCTTTAGCCTCTCCTG**	This study
*rpoB*-qPCR-FW	**TGCTGTTGAAATGGTTCCTG**	68
*rpoB*-qPCR-RV	**CGGTTGGCATCATCATTTTC**	68
*prdR*-upstream FW	**TATAAATAAGTTTTGATGAGGTACTAC**	This study
*prdR*-downstream RV	**AGACACATCTATTTTGTATTGGTC**	This study
*prdB*-upstream FW	**CGTTCCAATAACACCTCC**	This study
*prdB*-downstream RV	**TAACCGTGTAGGAGAAGGG**	This study
gBlock for TargeTron		
*prdR*_gBlock	TTCCCCTCTAGAAAAAAGCTTATAATTATCCTTATAATGCGCTCTTGTGCGCCCAGATAGGGTGTTAAGTCAAGTAGTTTAAGGTACTACTCTGTAAGATAACACAGAAAACAGCCAACCTAACCGAAAAGCGAAAGCTGATACGGGAACAGAGCACGGTTGGAAAGCGATGAGTTACCTAAAGACAATCGGGTACGACTGAGTCGCAATGTTAATCAGATATAAGGTATAAGTTGTGTTTACTGAACGCAAGTTTCTAATTTCGATTCATTATCGATAGAGGAAAGTGTCTGAAACCTCTAGTACAAAGAAAGGTAAGTTAACAAGAGCGACTTATCTGTTATCACCACATTTGTACAATCTG	This study
*prdB*_gBlock	TTCCCCTCTAGAAAAAAGCTTATAATTATCCTTACTCTACGAGTTCGTGCGCCCAGATAGGGTGTTAAGTCAAGTAGTTTAAGGTACTACTCTGTAAGATAACACAGAAAACAGCCAACCTAACCGAAAAGCGAAAGCTGATACGGGAACAGAGCACGGTTGGAAAGCGATGAGTTACCTAAAGACAATCGGGTACGACTGAGTCGCAATGTTAATCAGATATAAGGTATAAGTTGTGTTTACTGAACGCAAGTTTCTAATTTCGATTTAGAGTCGATAGAGGAAAGTGTCTGAAACCTCTAGTACAAAGAAAGGTAAGTTACGGAACTCGACTTATCTGTTATCACCACATTTGTACAATCTG	This study

a Boldface font indicates region in primer with sequence similarity to C. difficile.

### Animal infections.

All animal experiments under protocol M1700053 were reviewed and approved by the Institutional Animal Care and Use Committee of Vanderbilt University. Procedures were performed according to the institutional policies, the Animal Welfare Act, NIH guidelines, and the American Veterinary Medical Association guidelines on euthanasia.

For the mouse model of CDI, age-matched adult (7 to 10 weeks old) male C57BL/6 mice were either purchased from the Jackson Laboratory (wild type) or bred in-house (S100A9^−/−^) (40). The cefoperazone mouse model of CDI used in this study is in accordance with the protocol previously described (34). Briefly, mice were treated with 0.5 mg/ml cefoperazone in their drinking water for 5 days, followed by 2 days of recovery with normal drinking water. For infections, mice were inoculated via oral gavage with 1 × 10^5^ spores in 100 μl of water of either a single strain of C. difficile or a 1:1 mixed inoculum in competitive infections. Prior to infection, mice were confirmed to be C. difficile negative via plating. The infection was allowed to persist for up to 4 days before mice were either euthanized or started on vancomycin in a relapse model (65). Here, on day 4 postinfection, mice were provided 0.4 mg/ml vancomycin in their drinking water for 5 days. Vancomycin was then removed and mice were provided normal drinking water for up to 5 days while weights and fecal burdens were monitored. Mice were euthanized by day 14 postinfection.

To determine bacterial burdens, fecal pellets or colon contents were weighed and homogenized in phosphate-buffered saline (PBS) and then serially diluted plated on taurocholate cycloserine cefoxitin fructose agar (TCCFA) for enumeration as CFU per gram of feces. For competitive infections, dilutions were also plated on TCCFA plus Linc to enumerate the abundance of the mutant strains.

### RNA-seq.

C. difficile was grown anaerobically in triplicates in BHIS broth overnight. The overnight culture was diluted 1:100 in calprotectin medium (CPM), composed of a 1:1 ratio of BHIS and calprotectin buffer (20 mM Tris [pH 7.5], 100 mM NaCl, 3 mM CaCl_2_) with and without recombinant calprotectin (CP). CP preparation was as described previously (66) and here was added to a final concentration of 0.35 mg/ml. The cultures were incubated at 37°C to an OD_600_ of 0.3. Upon reaching this density, the bacterial cells were stored at −80°C in a 1:1 solution of acetone and ethanol in a volume equal to the culture volume. To extract RNA, samples were thawed on ice, pelleted, and resuspended in LETS buffer (1 M LiCl, 0.5 M EDTA, and 1 M Tris [pH 7.4]). Cells were transferred to lysing matrix B tubes (MP Biomedicals) and lysed using a FastPrep-24 (MP Biomedicals) bead beater for 45 s at 6 m/s. Samples were then incubated at 55°C for 5 min and pelleted by centrifugation. The supernatant was transferred to a tube containing 1 ml TRIzol reagent (Thermo Scientific) and 200 μl chloroform. The mixture was vortexed and then centrifuged at maximum speed for 15 min. The aqueous upper layer was then transferred to a fresh tube with 1 ml isopropyl alcohol to precipitate RNA. RNA was pelleted and then washed with 200 μl 70% ethanol. Samples were air dried for 1 min and then resuspended in 100 μl RNase-free water. DNA contamination was removed using RQ1 DNase I (Promega) according to the manufacturer’s instructions. After DNase treatment, RNA was further purified using the RNeasy miniprep RNA cleanup kit (Qiagen) according to the manufacturer’s instructions. RNA concentration was determined using the Synergy 2 with Gen 5 software (BioTek).

RNA-seq library construction and sequencing were performed by HudsonAlpha. Concentration was determined using the Quant-iT RiboGreen RNA assay (Thermo Scientific), and integrity was visualized using an RNA 6000 nano chip (Agilent) on an Agilent 2100 Bioanalyzer (Agilent Technologies, Inc., Santa Clara, CA). RNA was normalized to 500 ng of total RNA for each sample, and the rRNA was removed using a Ribo-Zero rRNA Removal kit (Illumina). Directly after rRNA removal, the RNA was fragmented and primed for first-strand synthesis using the NEBNext First Strand synthesis module (New England BioLabs, Inc.) followed by second-strand synthesis using NEBNext Ultra Directional Second Strand synthesis kit. Library preparation was achieved using NEBNext DNA Library Prep Master Mix set for Illumina with minor modifications. poly(A) addition and custom adapter ligation were performed following end repair. Postligated samples were individually barcoded with unique in-house Genomic Services Lab (GSL) primers and amplified through 12 cycles of PCR. Library quantity was assessed by a Qubit 2.0 Fluorometer (Invitrogen), and quality was determined using a DNA High Sense chip on a Caliper Gx (PerkinElmer). Final quantification of the complete libraries for sequencing applications was measured using the quantitative PCR (qPCR)-based KAPA Biosystems Library Quantification kit (Kapa Biosystems, Inc.). Libraries were diluted to 12.5 nM and pooled equimolar prior to clustering. Paired-end (PE) sequencing was performed on an Illumina HiSeq 2500 sequencer (Illumina, Inc.).

RNA-seq analysis was performed by HudsonAlpha using their unique in-house pipeline. Briefly, quality control was performed on raw sequence data from each sample using FastQC (Babraham Bioinformatics). Curated raw reads were imported into the data analysis platform, Avadis NGS (Strand Scientifics), and mapped to the reference C. difficile R20291 genome. Aligned reads were filtered according to various criteria to ensure the highest read quality. Replicate samples were grouped and quantification of transcript performed using trimmed means of M-values (TMM) as the normalization method. Differential expression of genes was calculated using fold change (using default cutoff of ≥±2.0) observed between conditions, and the *P* value of the differentially expressed gene list was estimated by Z-score calculations determined by Benjamini Hochberg false-discovery rate (FDR) correction of 0.05.

### Gene categories.

C. difficile genes with significantly different expression levels (>3-fold or <3-fold change) as determined by RNA-seq were manually assigned categories based on predicted or known functions. Predicted functions were determined both by using NCBI protein BLAST to search for homology to known protein domains and by comparing genome annotations to gene ontology (GO) categories.

### Nanostring.

**(i) *In vitro* RNA isolation.** Three independent WT C. difficile colonies were grown overnight in BHIS broth. The overnight cultures were diluted 1:50 into CPM with or without calprotectin at 0.32 mg/ml and incubated at 37°C until they reached an OD_600_ of approximately 0.3. Bacteria were pelleted and then stored in a 1:1 solution of acetone and ethanol at −80°C. RNA was isolated as described above (see “RNA-seq”).

**(ii) *In vivo* RNA isolation.** Wild-type C57BL/6J mice were either vehicle treated or infected (*N* = 10) with WT C. difficile as described above. On day 2 postinfection, mice were euthanized, and their ceca were isolated and then flash frozen in liquid nitrogen. RNA was isolated using the RNeasy PowerMicrobiome kit (Qiagen) according to the manufacturer’s instructions with the noted changes. Instead of a vortexer, samples were added to lysing matrix A tubes (MP Biomedicals). These were then moved to the bead beater for 40 s at 4 m/s, followed by a 5-min rest, and then 20 s at 6 m/s. Samples were eluted with 100 μl of RNase-free water and then DNase treated as described above (see “RNA-seq”).

**(iii) Nanostring processing.** One hundred nanograms of RNA from each sample (both from *in vitro* and *in vivo* isolation) was added to PCR strips to a final volume of 5 μl. Next, 8 μl of the Reporter CodeSet (Nanostring Technologies, Inc.) (see Table S2) in hybridization buffer was added to each sample followed by 2 μl of the Capture ProbeSet. Contents were mixed then immediately placed in a thermocycler preheated to 65°C. Hybridizations were incubated for 16 h, and then placed on ice. Samples were processed on the FLEX analysis system according to the manufacturer’s instructions.

Data were processed using nSolver software (Nanostring technologies). Target transcript abundances were normalized to the geometric mean of *rpoA* transcript abundance. All analyzed target genes were confirmed to only be found above the minimum threshold in the infected mice and not the mock-treated mice; however, background subtraction was not performed to minimize artifacts from low-abundance transcripts as per the manufacturer’s recommendations. Fold change was calculated by comparing the mean normalized abundance of C. difficile transcripts from infected mice to the abundance of transcripts *in vitro*. To determine significantly different transcript abundances, natural-log-transformed data were analyzed using a 2-way analysis of variance (ANOVA) followed by Sidak correction for multiple comparisons at an alpha of 0.05.

### RNA isolation.

To determine relative transcript abundance in response to calprotectin, C. difficile was grown anaerobically in BHIS broth overnight. The overnight cultures were diluted 1:100 into TY (30 g/liter tryptone, 20 g/liter yeast extract) calprotectin medium (TCPM), composed of a 1:1 ratio of TY and calprotectin buffer with and without purified CP at 0.5 mg/ml. To determine the specific effects of Zn and Mn sequestration on proline gene transcription, the following reagents were added: l-proline, 30 mM, CP ΔSI (66, 67), 1 mg/ml; CP ΔSI/ΔSII (66), 0.5 mg/ml; ZnCl_2_, 42 μM or 150 μM, MnCl_2_, 42 μM. CP ΔSI was added at double the concentration as CP to equalize the numbers of metal-binding sites (i.e., one in CP ΔSI and two in CP). As CP ΔSI/ΔSII does not contain metal-binding sites, it was added at the same concentration as CP. Bacterial cultures were grown at 37°C until they reached late exponential phase, when they were then centrifuged and stored at −80°C in a 1:1 mix of acetone and ethanol until further processing. To isolate RNA, pellets were thawed and centrifuged. The supernatant was removed, and the pellets were resuspended in LETS buffer and then transferred to lysing matrix B tubes. Tubes were placed into a bead beater set for 6 m/s for 40 s. Samples were then heated to 55°C for 5 min. Lysed bacteria were centrifuged, and RNA from the supernatant was extracted using an RNeasy miniprep RNA cleanup kit. RNA was eluted with 100 μl of RNase-free water and then treated with RQ1 DNase according to the manufacturer’s instructions.

To determine relative transcript abundance in response to *N*,*N*,*N*′,*N*′-tetrakis(2-pyridinylmethyl)-1,2-ethanediamine (TPEN) or 2,2-dipyridyl, C. difficile was grown anaerobically in BHIS broth overnight. The overnight cultures were diluted 1:100 into TY medium and grown as described above. The following reagents were used: l-proline, 30 mM; TPEN, 75 μM; ZnCl_2_, 75 μM; 2,2-dipyridyl, 50 μM. RNA was isolated as described above.

### qPCR.

One microgram of RNA was reverse transcribed by M-MLV reverse transcriptase (Fisher Scientific) in the presence of RNase inhibitor (Promega) and random hexamers (Promega). Newly synthesized cDNA was diluted 1:20 in water and used in reverse transcription-quantitative PCR (qRT-PCR) using iQ SYBR green supermix (Bio-Rad). Amplification was achieved using a 3-step melt curve program on a CFX96 qPCR cycler (Bio-Rad). Target gene expression was normalized to the *rpoB* housekeeping gene using the threshold cycle (ΔΔ*C_T_*) method (for primers, see Table 2 and reference 68).

### Growth curves.

The appropriate bacterial strains were first grown on BHIS agar and then grown overnight in BHIS broth. The overnight cultures were diluted 1:100 in fresh BHIS broth and incubated at 37°C for 6 h. The OD_600_ values of the cultures were normalized to the same density, and then the cultures were diluted 1:50 in fresh TY medium containing 30 mM l-proline. For each condition, 200 μl of inoculated medium was transferred to a single well in a 96-well clear round-bottom plate. Each condition was performed in experimental triplicates, with the densities averaged for each datum point. Each condition was also repeated independently at least 3 times. The 96-well plate was covered with a breathable film and then placed into an Epoch 2 plate reader (Biotek) within the anaerobe chamber. Samples were incubated at 37°C with double orbital continuous shaking, with the OD_600_ measured and recorded every 30 min.

### 5-Aminovalerate measurements.

For *in vitro* 5-aminovalerate measurements, the WT, *prdB*::*CT*, or *prdR*::*CT* strain was first grown overnight in BHIS broth. Cultures were then diluted 1:100 in TY with 30 mM l-proline. When appropriate, sodium selenite (10 μM), ZnCl_2_ (75 μM), and TPEN (75 μM unless otherwise indicated) were added to the medium at the start of growth. At various time points, 1 ml of medium was removed from each sample and centrifuged at high speed. The supernatants were then transferred to 0.22-μm filter spin columns to remove bacteria. Flow through was collected and stored at −20°C until further analysis.

For 5-aminovalerate measurements from fecal contents, fecal pellets were recovered from mice in preweighed tubes and placed in 1 ml of distilled water. Feces were homogenized and then centrifuged at high speed. The supernatants were transferred to a 0.22-μm spin column. Flow through was collected and stored at −20°C until further analysis.

Samples for 5-aminovalerate quantification were analyzed as previously described (69). Heptafluorobutyric acid (HFBA) (Sigma) was added to each sample to 75 mM as an ion-pairing agent. Samples were analyzed on a Thermo TSQ Quantum Ultra with an electrospray ionization (ESI) source interfaced to a Waters Acquity ultraperformance liquid chromatography (UPLC) system. Analytes were separated by gradient high-performance liquid chromatography (HPLC) with Agilent Poroshell 120 C_18_ (3.0 mm by 50 mm, 2.7 μm) and a Phenomenex SecurityGuard C_18_ (3.2 mm by 8 mm) cartridges at a flow rate of 0.3 ml/min using 10 mM HFBA in water and 10 mM HFBA in acetonitrile as the A and B mobile phases, respectively. The gradient was held at 0% B for 1 min and then ramped to 100% B over the next 8 min. The column was washed with 100% B for 3 min and then equilibrated to 0% B for 3 min. 5-Aminovalerate was analyzed by multiple-reaction monitoring in negative ionization mode at an *m/z* transition for 5-aminovalerate (118.1 to 55.1) using a collision energy of 16 eV. Skimmer offset and tube lens voltages were determined empirically before each set of samples was run. Quantification was performed by comparing analyte area under the concentration-time curve (AUC) values to those of an external calibration line of 5-aminovalerate at 0, 1, 3, 10, 30, 100, 300, and 1,000 μM.

### NAD/NADH ratio.

WT C. difficile and the *prdB*::CT mutant were cultured overnight in 5 ml BHIS broth. These cultures were then diluted 1:100 into TY medium containing 30 mM proline. TPEN and ZnCl_2_ were added at 75 μM final concentration. Cultures were incubated at 37°C until an OD_600_ of 0.270 was reached, and then 2 ml of the culture was centrifuged to pellet the bacteria. Cells were washed twice with sterile water and then resuspended in B-per reagent (Thermo Fisher) with 500 μg/ml lysozyme. Samples were incubated at room temperature for 15 min and then placed on ice for the remainder of processing. Samples were next sonicated for 12 s at an amplitude of 40 and then again for 10 s at an amplitude of 100. Samples were then transferred to a 0.5-ml protein spin column (10K molecular weight cutoff) and centrifuged. The flowthrough was transferred to a cryovial and stored at −80°C. To measure the NAD/NADH ratio, samples were thawed then processed using the NAD/NADH assay kit (ab65348; Abcam) according to the manufacturer’s instructions. OD_430_ was measured for 4 h, with readings every 20 min following shaking for 5 s. The NAD/NADH ratio was calculated using a standard curve prepared according to the manufacturer’s instructions.

### Inductively coupled plasma mass spectrometry.

To determine intracellular elemental levels, C. difficile was grown overnight in BHIS broth from a plate. Experimental medium was composed of TY with or without TPEN (75 μM) and sodium selenite (10 μM). C. difficile was diluted 1:100 from the overnight culture into the experimental medium. Cultures were incubated at 37°C until they reached an OD_600_ of approximately 0.6. For all subsequent procedures, metal-free pipette tips and tubes were used. Next, 1 ml of each culture was transferred to a preweighed tube, centrifuged, and washed twice with Ultrapure water. The weight of the final bacterial pellet, with supernatant removed, was recorded. To prepare for ICP-MS, bacterial pellets were incubated at 60°C overnight in 200 μl Optima nitric acid and 50 μl 30% hydrogen peroxide. Following digestion, 1 ml of Ultrapure water was added.

To determine elemental levels in feces, fecal pellets were recovered from mice in preweighed tubes and placed in 1 ml of Ultrapure water. Feces were homogenized and then centrifuged at high speed. The supernatants were transferred to a 0.22-μm spin column. The flowthrough was collected and digested for elemental analysis as above.

Elemental quantification was performed using an Agilent 7700 ICP-MS (Agilent) attached to a Teledyne CETAC Technologies ASX-560 autosampler (Teledyne CETAC Technologies). The following settings were fixed for the analysis: cell entrance, −40 V; cell exit, −60 V; plate bias, −60 V; octP bias, −18 V; collision cell helium flow, 4.5 ml/min. Optimal voltages for Extract 2, Omega Bias, Omega Lens, OctP RF, and Deflect were determined empirically before each sample set was analyzed. Element calibration curves were generated using ARISTAR ICP standard mix (VWR). Samples were introduced by a peristaltic pump with 0.5-mm-internal-diameter tubing through a MicroMist borosilicate glass nebulizer (Agilent). Samples were initially taken up at 0.5 rps for 30 s followed by 30 s at 0.1 rps to stabilize the signal. Samples were analyzed in spectrum mode at 0.1 rps, collecting three points across each peak and performing three replicates of 100 sweeps for each element analyzed. Sampling probe and tubing were rinsed for 20 s at 0.5 rps with 2% nitric acid after every sample. Data were acquired and analyzed using the Agilent Mass Hunter Workstation Software version A.01.02. Elemental data were normalized to the weight of the bacterial pellet or fecal supernatant.

### Proteomics.

To quantify intracellular peptide abundances, C. difficile was grown overnight in BHIS broth from a plate. Experimental medium was composed of TY with or without TPEN (75 μM) and sodium selenite (10 μM). C. difficile was diluted 1:100 from the overnight culture into the experimental medium. Cultures were incubated at 37°C until they reached an OD_600_ of approximately 0.6. At this point, 5 ml of sample was centrifuged, and the supernatant was removed. To lyse cells, the bacterial pellets were incubated with 400 μl B-per reagent (Thermo Fisher) and 0.2 mg lysozyme at room temperature for 15 min. Afterwards, to each sample, 1 ml of an 80% acetonitrile, 5% formic acid, and 15% water solution was added. From here on, samples were stored on ice. Samples were then sonicated, first at power amplitude 40 for 12 s and then at amplitude 100 for 10 s. These were stored at −20°C until further processing.

Peptide quantification involved preparing 20 μg of protein using S-trap (Protifi, Farmingdale, NY) cleanup and trypsin digestion according to the manufacturer’s recommended protocol. One microgram of the resulting peptide mixture was injected and subsequently resolved on a 100-μm by 20-cm self-packed reverse-phase (Phenomonex, Jupiter 3 μm, 300 Å) HPLC column positioned for nanoelectrospray directly in a QExactive-plus mass spectrometer (Thermo Fisher). Over the course of an 80-min aqueous-to-organic gradient, data were collected using a parallel reaction monitoring (PRM) strategy across a set of 24 masses followed by a single full MS scan; 2.0 *m/z* width isolation PRM scans were performed at 17,500 resolution with a 1e5 automatic gain control (AGC) target and a normalized collision energy (NCE) of 27. Full MS scans were from 300 to 1,500 *m/z* at 70,000 resolution and 3e6 AGC target value. Resulting data were analyzed using Skyline (skyline.ms/) software package for both visualization and quantification.

### Data availability.

RNA-sequencing data can be found at the NCBI Gene Expression Omnibus (https://www.ncbi.nlm.nih.gov/geo/) under accession numbers GSE135912, GSM4037804, GSM4037805, GSM4037806, GSM4037807, GSM4037808, and GSM4037809.
