# Supplementary figures and images for: The Immune Protein Calprotectin Impacts Clostridioides difficile Metabolism through Zinc Limitation
Source: mBio. 2019 Nov 19;10(6):e02289-19. doi: 10.1128/mBio.02289-19 (PMC6867894; doi:10.1128/mBio.02289-19)

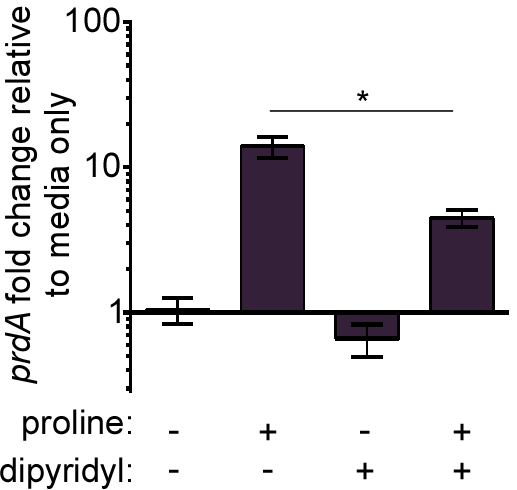

Supplement: FIG S1 [file mBio.02289-19-sf001.tif]

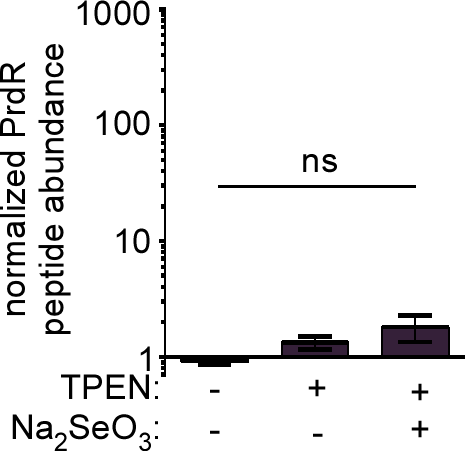

Supplement: FIG S2 [file mBio.02289-19-sf002.tif]
